# Melatonin: From Pharmacokinetics to Clinical Use in Autism Spectrum Disorder

**DOI:** 10.3390/ijms22031490

**Published:** 2021-02-02

**Authors:** Sébastien Lalanne, Claire Fougerou-Leurent, George M. Anderson, Carmen M. Schroder, Tali Nir, Sylvie Chokron, Richard Delorme, Bruno Claustrat, Eric Bellissant, Solenn Kermarrec, Patricia Franco, Laure Denis, Sylvie Tordjman

**Affiliations:** 1 Experimental and Clinical Pharmacology Department, CHU Rennes, 35033 Rennes, France; claire.fougerou@chu-rennes.fr (C.F.-L.); eric.bellissant@univ-rennes1.fr (E.B.); 2 Inserm, CIC 1414 (Clinical Investigation Center), 35033 Rennes, France; 3 Child Study Center, Yale University School of Medicine, New Haven, CT 06520, USA; george.anderson@yale.edu; 4 Department of Child and Adolescent Psychiatry, Strasbourg University Hospitals, 67000 Strasbourg, France; carmen.schroder@chru-strasbourg.fr; 5 CNRS UPR 3212, Institute for Cellular and Integrative Neurosciences, 67000 Strasbourg, France; 6 Sleep Disorders Center & CIRCSom (International Research Center for ChronoSomnology), Strasbourg University Hospital, 67000 Strasbourg, France; 7 Neurim Pharmaceuticals Ltd., Tel Aviv 69710, Israel; talin@neurim.com; 8 Integrative Neuroscience and Cognition Center (INCC), CNRS UMR 8002 and University of Paris, 75006 Paris, France; sylvie.chokron@gmail.com; 9 Child and Adolescent Psychiatry Department, Robert Debré Hospital and University of Paris, 75019 Paris, France; richard.delorme@aphp.fr; 10 Laboratoire d’Hormonologie, Inserm U846, Centre de Médecine Nucléaire, Hospices Civils de Lyon, 69677 Bron, France; bruno.claustrat@sfr.fr; 11 School of Medicine of Rennes, University of Rennes 1, 35000 Rennes, France; 12 Pôle Hospitalo-Universitaire de Psychiatrie de l’Enfant et de l’Adolescent (PHUPEA), Université de Rennes 1 and Centre Hospitalier Guillaume-Régnier, 35000 Rennes, France; kermarrec@ch-guillaumeregnier.fr (S.K.); l.denis@ch-guillaumeregnier.fr (L.D.); 13 Pediatric Sleep Unit, Inserm U1028, University of Lyon 1, 69000 Lyon, France; patricia.franco@univ-lyon1.fr

**Keywords:** melatonin, circadian rhythm, pharmacokinetics, autism, autism spectrum disorder (ASD), autistic behavioral impairments, concentration-effect relationship, dose-response effect, tolerability, analytical variability

## Abstract

The role of melatonin has been extensively investigated in pathophysiological conditions, including autism spectrum disorder (ASD). Reduced melatonin secretion has been reported in ASD and led to many clinical trials using immediate-release and prolonged-release oral formulations of melatonin. However, melatonin’s effects in ASD and the choice of formulation type require further study. Therapeutic benefits of melatonin on sleep disorders in ASD were observed, notably on sleep latency and sleep quality. Importantly, melatonin may also have a role in improving autistic behavioral impairments. The objective of this article is to review factors influencing treatment response and possible side effects following melatonin administration. It appears that the effects of exposure to exogenous melatonin are dependent on age, sex, route and time of administration, formulation type, dose, and association with several substances (such as tobacco or contraceptive pills). In addition, no major melatonin-related adverse effect was described in typical development and ASD. In conclusion, melatonin represents currently a well-validated and tolerated treatment for sleep disorders in children and adolescents with ASD. A more thorough consideration of factors influencing melatonin pharmacokinetics could illuminate the best use of melatonin in this population. Future studies are required in ASD to explore further dose-effect relationships of melatonin on sleep problems and autistic behavioral impairments.

## 1. Introduction

### 1.1. Melatonin: Origin and Production

**Figure 1:**
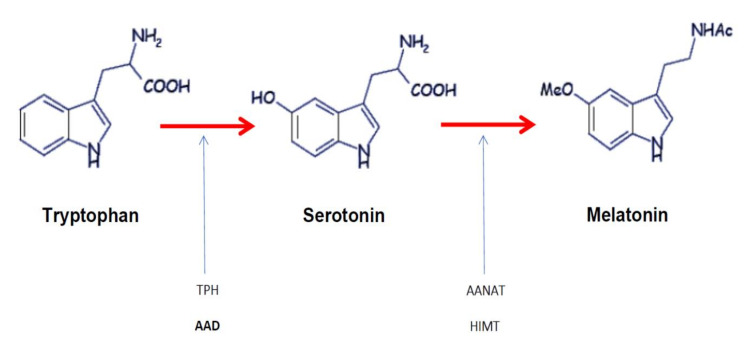
Melatonin synthetic pathway. Tryptophan is converted to 5-OH tryptophan by tryptophan 5-hydroxylase (TPH) and to serotonin by aromatic amino acid decarboxylase (AAD). Then, serotonin is converted to melatonin by the action of arylalkylamine N-acetyltransferase (AANAT) and hydroxyindole O-methyltransferase (HIMT) (based on Tordjman et al. [4]).

Melatonin or 5-methoxy-*N*-acetyl-tryptamine is a neurohormone that was isolated and named in 1958. Its name comes from its effect on frog skin pigmentation (melanin) and its structural similarity to serotonin. Its secretion is inhibited by light and regulated by the circadian clock located in the hypothalamic suprachiasmatic nuclei. Melatonin is synthetized from an essential amino acid, tryptophan [1]. Tryptophan undergoes three chemical steps before being transformed into melatonin (see Figure 1). In humans, this neurohormone is mainly produced in the pineal gland, gastrointestinal tract, and retina, but only melatonin secretion by the pineal gland and retina follows a typical circadian rhythm [1]. At the onset of darkness, reduced retinal input leads to the disinhibition of enzymes responsible for melatonin synthesis [2]. This increased nighttime synthesis results in peak nocturnal plasma concentrations of about 80 to 120 pg/mL between 2 and 4 am, levels decrease until daylight onset, with low (10–20 pg/mL) concentrations being observed during the daytime [3]. 

### 1.2. Melatonin: Physiological Mechanisms and Properties

**Figure 2:**
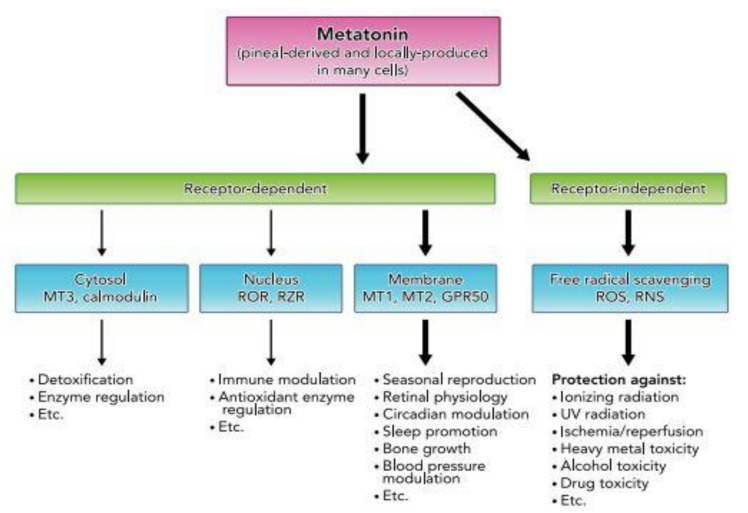
The two main melatonin physiological pathways in humans. The receptor-dependent and receptor-independent pathways are shown with their relevant families of receptors and their associated pleiotropic physiological effects (based on Reiter et al. [1]).

Melatonin acts through two major pathways: a receptor-mediated pathway (membrane, cytosolic, and nuclear receptors) and a receptor-independent pathway (see Figure 2). The receptor-mediated pathway is characterized by the activation of two types of membrane-specific receptors: the ML1 receptors, including MT1 (or Mel1a) and MT2 (or Mel1b) receptors, and the ML2 receptors, also called MT3 receptors. MT1 and MT2 are high-affinity receptors for melatonin with 60% homology, and their activation leads to an inhibition of the adenylate cyclase in target cells. These G-protein-coupled receptors have mainly a role in the regulation of vigilance states, sleep/wake rhythms, and bone mass regulation [1,4]. The MT3 receptor is a cytosolic receptor with low affinity for melatonin and has been shown to be a quinone reductase whose main role is detoxication. The third receptor-dependent pathway concerns nuclear receptors-retinoid orphan receptors (ROR) or retinoid Z receptors (RZR)-which may act in immune modulation and antioxidant enzyme regulation. Contrary to previous assertions, melatonin may be present at the surface and in numerous cells [1,5]. On the other hand, the receptor-independent action of melatonin consists of its directly detoxifying reactive oxygen and nitrogen species (ROS, RNS) [1]. Given its involvement in many pathophysiological mechanisms, supplementation with melatonin and/or its derivatives has been the subject of numerous trials as a drug or dietary supplement. Rivara and collaborators [6] summarized applications being currently studied in vitro or in vivo. Melatonin is especially recognized for its potential in treating sleep problems, its oncostatic effect and reduction of cancer side effects, hypertension, gastric disease, and diabetes (melatonin may reduce insulin levels). Its antioxidant action and immune system enhancement effects might contribute to some of the reported beneficial effects [4,6].

### 1.3. Melatonin: Supplementation in Central Nervous System (CNS) Disorders

The impact of melatonin in central nervous system (CNS) disorders has been the most investigated since its discovery. Sleep disorders have been widely studied. Among other promising melatonin effects, benefits of “melatherapy” are described in neurodegenerative diseases through mechanisms contributing in vitro to the induction of autophagy processes; autophagy may prevent the accumulation of misfolded proteins in Alzheimer’s or Parkinson’s diseases [7]. The analgesic effects of melatonin on chronic pain have also been reported in humans and animal models; animal models are of particular interest when studying CNS disorders and the neurobehavioral genetic factors possibly involved in these disorders [8,9]. Furthermore, melatonin administration has been shown to be efficient in somatoform disorders, including fibromyalgia, irritable bowel syndrome, functional dyspeptic syndrome, as well as temporo-mandibular pain disorders [10,11,12,13,14,15,16,17,18].

Currently, four melatonin medicines or derivates are approved with the following CNS indications: (a) a prolonged-release (PR) melatonin is approved by the EMA (European Medicines Agency) for the indication of “relief of primary insomnia characterized by poor quality of sleep in patients aged 55 or over” [19]; (b) a high-affinity MT2 receptor agonist (ramelteon) is approved in United States, Indonesia, and Japan for the treatment of insomnia in adults [20]; (c) a MT1/MT2 nonselective melatonin receptor agonist (tasimelteon) is approved by the FDA (Food and Drug Administration) for the treatment of Non-24-hour Sleep-Wake Disorder (Non-24 or N24SWD, a body endogenous clock desynchronization disorder) [6,21] and sleep-wake disorder in Smith–Magenis syndrome associated with altered diurnal melatonin secretion [4]; and (d) a MT1 and MT2 receptor agonist and serotonin receptor antagonist (agomelatine) is recognized for the treatment of major depressive disorder in adults [22].

Since 2015, PR melatonin has received in France a temporary recommendation for use (TRU) with a follow-up protocol in children aged 6 to 18 years treated for sleep–wake disorders associated with developmental disorders and/or neurogenetic diseases such as Rett’s syndrome, Smith–Magenis syndrome, Angelman’s syndrome, tuberous sclerosis, or autism spectrum disorder (ASD). It is noteworthy that this TRU is the only one in the world to authorize melatonin administration in children with ASD. However, a positive recommendation was given on September 2018 by the European Medicines Agency (EMA) under Pediatric Use Marketing Authorization (PUMA) for the use of a PR melatonin formulation for the treatment of insomnia in children and adolescents aged 2–18 years with ASD and/or Smith–Magenis syndrome [23,24].

Although melatonin has been widely studied and endogenous blood concentrations are well documented, there are limited data on melatonin pharmacokinetics (PK) and pharmacokinetics-pharmacodynamics (PK-PD) relationships in humans, as well as the factors that influence PK. The main objective of this article is to investigate the impact of PK variability on melatonin bioavailability and its therapeutic and possible side effects in individuals with ASD. In this context, factors influencing exposure to endogenous and exogenous melatonin in healthy individuals are reviewed, as well as melatonin PK properties according to the melatonin administration route and formulation type. Then, melatonin pharmacodynamics in individuals with ASD are discussed, especially with regard to dose-effect and concentration-effect relationships for melatonin supplementation, including optimal daily dose and formulation type in ASD. Finally, the effects of sampling and analytical method strategies on the measurement of melatonin concentrations in healthy and ASD individuals are examined.

## 2. Melatonin Variability Factors and Pharmacokinetics in Healthy Individuals

### 2.1. Endogenous Melatonin

Melatonin is released by the pineal gland, and its blood levels as well as urinary excretion rates of its principle metabolite 6-sulphatoxymelatoninare representative of pineal gland activity [25]. The variability of plasma concentrations of endogenous melatonin is significant in the general population, both during nighttime (peak period) and daytime (low period) [26]. Studies have demonstrated both inter- and intraindividual variability, thus enabling the identification of endogenous variability factors.

Among the main inter- and intraindividual variability factors, age plays a significant role in melatonin secretion. Melatonin secretion rhythm is typically established around 3 months of age and undergoes changes throughout life as follows: plasma melatonin levels in children are elevated compared to older individuals, and a gradual age-related decline in melatonin production occurs beginning around 20–30 years of age [27,28]. Cavallo et al. [29] confirmed previous studies [27,30] reporting a significant decrease of the nocturnal melatonin peak with puberty based on Tanner stages of puberty, with a maximum value of 175 pg/mL at 5–7 years of age. A significantly shorter elimination half-life (T_1/2_) is also observed in prepubertal children [31]. Furthermore, lower nocturnal peak levels in salivary melatonin were found in older healthy individuals, with a decrease in the circadian rhythms of melatonin beginning around 40 years of age [32].

The role of sex variability has also been confirmed in a recent prospective clinical trial [33] with a significant difference between male and female plasma melatonin Area Under Curve (AUC) (N = 32, age-matched groups, 642 ± 47 pg·h/mL for men, vs. 937 ± 104 pg·h/mL for women, *p* = 0.016), but not in the timing of melatonin onset and offset. The findings agree with prior results [34], observing higher endogenous melatonin levels in females than in males. This is not due to a difference in Body Mass Index. Although sex differences of endogenous melatonin metabolism, melatonin circadian profiles, and the regulation of melatonin secretion (positive for estradiol and negative for testosterone) have been described, the metabolite excretion rate was not significantly different between males and females [33].

With regard to exogenous factors, the role of seasonal rhythms on melatonin secretion profiles needs to be better ascertained. Indeed, a circannual rhythm with a peak of melatonin secretion during the winter months has been shown in some small-scale studies in regions with a strong seasonal contrast in luminosity (such as the Kauppila et al. study conducted on 11 females [35]), but there is little evidence for such circannual rhythm in temperate latitudes [36]. Concerning these two well-identified factors (age and sex), it is worth mentioning that in populations matched on these criteria, endogenous melatonin shows less interindividual variability [33].

In addition, melatonin is not equally distributed and synthetized in different biological compartments with local levels of melatonin being higher than blood levels in bile, cerebrospinal fluid, or intracellular compartments [1,37]. We have little information about the role of these melatonin “pools” and the importance of membranous transporters such as the peptide transporters PEPT 1/2 and the organic anion transporter OAT3 that have been found recently to be involved in melatonin transport [38].

### 2.2. Exogenous Melatonin

It has been established that usual doses (1–12 mg) of exogenous melatonin administration reach concentrations 10 to 100 higher than endogenous peak values [4]. Furthermore, the large number of formulation types used, the limited number of PK studies and their variable designs and data, as well as the lack of consideration of factors causing a variation of endogenous melatonin levels warrants a review of current knowledge to optimize melatonin use in the future. Melatonin pharmacokinetics have been little studied in preterm neonates [39] or healthy children; thus, the following literature review concerns mostly healthy adults.

#### 2.2.1. General Pharmacokinetic Properties

Melatonin is poorly absorbed for all formulations, with bioavailability ranging from 2.5% to 33% [40,41] and with in vitro protein binding of 60% [42]. It undergoes substantial hepatic metabolism, particularly for oral formulations with high hepatic first pass effect [43,44], as evidenced in an increased ratio of metabolite to melatonin for oral preparations compared to IV. The combination of both poor absorption and substantial hepatic first pass metabolism explains much of the compound’s low bioavailability [44]. Animal studies show that hepatic melatonin metabolism mainly occurs through CYP1A2 and CYP2C19 (hydroxylation to 6-hydroxymelatonin) [43,45]. Then, 6-hydroxy-melatonin is sulfate conjugated to 6-sulfatoxymelatonin (6-SM), which represents 80% of melatonin’s metabolites [43]. Similar results from human studies are reported [42]. The 6-SM metabolite undergoes urinary excretion and is considered inactive [4]. However, there is a current lack of knowledge regarding melatonin metabolites in humans, and recent studies highlighted the existence of active metabolites [43].

Based on the Harpsøe et al. [40] review and subsequent studies, we identified 22 studies on melatonin in healthy volunteers. As previously indicated, these studies showed highly variable data that need to be matched according to the identified variability factors for endogenous melatonin (see above), the type of population studied, and especially according to the formulation and route of administration used. Among these 22 studies, only 11 studies specifying the formulation type allow the comparison of the main PK properties. These 11 studies are summarized in Table 1.

#### 2.2.2. Specific Pharmacokinetic Properties based on Melatonin Administration Route

##### Intravenous Injection

For intravenous (IV) administration, seven studies [41,44,46,47,48,49,50] included age-matched volunteers exposed from 0.005 to 100 mg of IV melatonin. Fourtillan et al. [47] studied differences between the Area Under Curve (AUC) in 12 males vs. females after IV administration of 25 µg of melatonin and found a significant difference (females: 364 ± 64 pg·h/mL, males: 255 ± 59 pg·h/mL) but no differences in clearance. These results are consistent with sex differences observed for endogenous melatonin. The AUC showed acceptable inter-experimental linearity, with a decrease of intra-study variability when selection was made based on age and sex. For example, Andersen et al. [48] found an AUC 0-∞ between 7 × 10^6^ and 18 × 10^6^ pg·h/mL in 12 healthy male volunteers from 20 to 40 years old who were administrated the same 10 mg dose of IV melatonin, whereas DeMuro et al. [46] found an AUC between 1.6 × 10^6^ and 2.1 × 10^6^ pg·min/mL for six male and six female healthy volunteers (2 mg dosage) with the same age range. Di et al. [44] reported substantial inter-individual variations in peak melatonin concentrations after 20 and 500 µg doses (from 480 × 10^3^ to 9.2 × 10^6^ pg/mL) but studied a small sample (*n* = 4). Taken together, the IV data indicate that age, sex, and absorption play a significant role in exposure to melatonin. IV administration tends to reduce interindividual variation, as it bypasses the hepatic first-pass effect.

##### Oral Immediate-Release formulations

Melatonin PK was examined for oral melatonin supplementation in a total of seven studies of immediate-release (IR) formulations in healthy volunteers [42,46,47,48,51,52,53]. IR preparations include capsules, tablets, and oral solutions. Overall, PK parameters, such as AUC, C_max_, and T_max,_ showed higher variability than for IV melatonin. It seems to be very difficult to compare the data from the different studies because some parameters were not consistent across studies, such as age, sex ratio, healthy volunteers recruited, formulation type, time of melatonin administration, and measuring period.

For tablets, two studies explored PK data in age-comparable populations [46,51] of healthy volunteers. One of these trials established 15% bioavailability in a randomized crossover bioavailability study [46]. AUC and C_max_ were highly variable, with a mean AUC coefficient of variation (CV) of 57% for oral administration vs. 26% for IV administration [46] and different nonlinear AUC between studies (from 237 × 10^3^ pg·h/mL for 2 mg to 1.2 × 10^6^ pg·h/mLfor 6 mg tablets). However, a T_max_ of 49 min was quite similar for dosages ranging from 2 to 6 mg, and mean T_1/2_ was similar in oral administration compared to IV administration, showing little variability in excretion.

In three studies [48,52,53] using IR capsules (0.3 to 240 mg) with comparable age volunteers, similar values for T_max_, T_1/2_, C_max_, and AUC were found. Andersen et al. reported a mean bioavailability of 2.5% with a range of 1.7 to 4.7% in a crossover study with young male adults.

Finally, in another study, 0.25 mg oral solution was administrated to 12 young healthy adults (six males and six females) [47]. This trial gave results consistent with endogenous melatonin PK properties. They found higher levels in females than in males (701 ± 645 pg·h/mL and 236 ± 107 pg·h/mL, respectively) and found also substantial interindividual variability, particularly in the female group. Unfortunately, this study did not provide endogenous melatonin levels. Mean T_max_ values appeared similar across subjects and were reduced compared to other formulations (23 min). This crossover study showed higher (12%), but variable (1 to 37%) bioavailability.

##### Oral Prolonged-Release

As melatonin is being secreted throughout the night and immediate-release (IR) formulations present only a short half-life (about 1 hour), prolonged-release (PR) formulations have been developed. However, very few PK data are available for the PR formulation, although this formulation is widely used. Only one healthy volunteers study is provided by the EMA: a study on eight adult male volunteers where basal melatonin rate PK parameters (24 h AUC, C_max_, T_max_ and “plateau time”) were compared to those observed after administration of 2 mg PR melatonin tablet given at the same hour [42]. Melatonin basal levels showed, as expected, variable levels from 150 to 1017 pg·h/mL and AUC after supplementation varied from 823 to 4478 pg·h/mL. These data suggest that PR formulations present the same range of exposure variability than other formulations. However, mean T_max_ appeared longer than for other formulations (96 min). Interestingly, plateau time (apparent half-life) was 5.1 ± 2h and was close to the plateau time measured in basal state (6.9 ± 1.7 h), showing a potential capacity to mimic melatonin secretion. As underlined by Williams et al. [54], bioavailability estimations for PR formulations (10–20%) are only based on IR ones. Difficulties in swallowing PR melatonin tablets led to a practical study examining the impact of tablet division [55]. Divided into halves, tablets appeared to preserve their PR properties whereas quarter-cutting and crunching resulted in a more IR profile.

#### 2.2.3. Pharmacokinetic Properties and Extrinsic Variability Factors

##### Interactions with Other Substances

A great number of substances may affect melatonin PK properties. Among the main external PK variability factors [56] that must be taken into account, tobacco consumption was assessed [57]. Whereas endogenous melatonin levels were not affected by smoking, melatonin levels after oral administration were significantly lower (*p* ≤ 0.02) when smoking than after abstinence, suggesting that CYP1A2 tobacco induction coupled with a supraphysiological level during supplementation can lead to lower melatonin levels. Oral contraceptive pills (OCP) intake may also modify melatonin PK properties by their inhibitory effect on CYP1A2, possibly contributing to the higher mean melatonin levels observed in females (despite the absence of difference in elimination rates between males and females) [33]. Many other drug interactions have been reported to modify melatonin metabolism [58]. Selective serotonin reuptake inhibitors (SSRIs) such as fluvoxamine [45,59,60] and some antibiotics such as quinolones, which are strong CYP1A2 inhibitors, are among the drugs that can lead to increased or over-exposure to melatonin.

##### Effects of Age, Sex, and Route of Administration on Melatonin PK

Age and sex are two major parameters causing variations in endogenous melatonin exposure, as previously described in this article, but these can also affect exogenous melatonin exposure. In addition, and as mentioned previously, exposure to exogenous melatonin is dependent on the route of administration, leading particularly to variable AUC and C_max_ values for the oral route. T_max_ and T_1/2_ parameters are more homogeneous with mean values of 55 and 49 min, respectively, regardless of the route of administration. It is noteworthy that only the PR formulation appears to have a similar exposure profile to endogenous melatonin. Bioavailability data are limited and variable due to the intense hepatic first-pass effect; the oral solution seems to have a better bioavailability than solid formulations. However, even in homogeneous populations selected to minimize the effect of these variability factors, data remain variable, suggesting the existence of additional variability factors. Further investigations are required to clarify this issue. By using cohorts that are relatively homogenous with respect to age and sex, it should be possible to reduce inter-individual variability and to determine more accurately the PK characteristics of, and any differences between, endogenous and supplemented melatonin [47].

##### Effect of Time of Melatonin Administration

**Figure 3:**
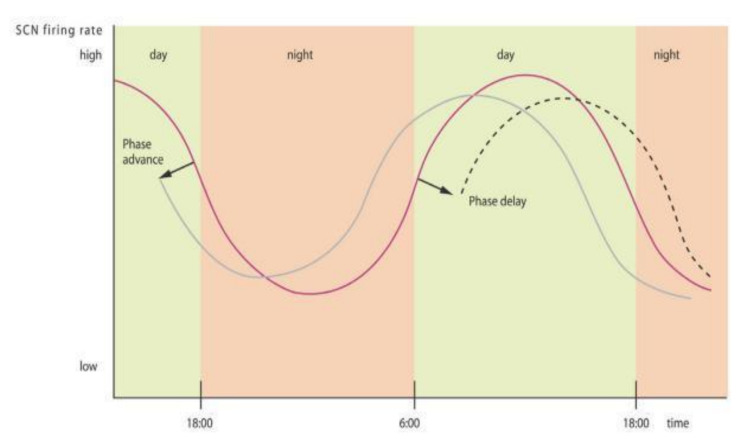
Melatonin chronobiological properties: exogenous melatonin administration modifies melatonin secretion by modulating suprachiasmatic (SCN) neuronal activity. A phase advance (solid gray line) is observed when melatonin is administered at the beginning of the night and a phase delay (dashed line) is seen when melatonin is administered in the morning (based on Grivas et al. [2]).

The time of administration of exogenous melatonin is a crucial variable to examine. Melatonin supplementation is known to modify endogenous secretion according to the phase response curve [45] and thus cannot be considered as simple as adding or triggering a negative feedback loop. Endogenous plasma melatonin profiles show an advanced profile after evening or night administration and a delayed profile following melatonin administration in morning or midday, with a turning point during the afternoon [61,62] (see Figure 3).

##### Effect of Genetic Variants on Melatonin Supplementation

Genotyping polymorphisms, particularly *CYP1A2* [45], which participates in the hepatic first pass-effect, could make it possible to select high or low oral melatonin metabolizers. Overall, a better knowledge of these factors would allow the use of physiologically based pharmacokinetic modeling approaches to anticipate melatonin exposure variations [63].

Studies of melatonin PK are of particular interest in autism spectrum disorder (ASD) in order to ascertain better the underlying physiological mechanisms involved in the melatonin abnormalities reported in ASD and to investigate to which extent melatonin supplementation could improve clinical signs, including sleep disorders observed in individuals with ASD [64,65]. Clinical studies of melatonin supplementation are reviewed and discussed below.

## 3. Melatonin and Autism Spectrum Disorder (ASD)

### 3.1. Relationships between Autistic Behavioral Impairments and Melatonin

Autism is defined in the DSM-5 [66] and ICD-10 [67] as impaired social communication and repetitive/restrictive behaviors or interests, with onset since the early developmental period. Many studies have reported abnormal melatonin secretion in autism, especially decreased nocturnal melatonin secretion and metabolites excretion as well as altered circadian rhythms of melatonin [68,69]. It is noteworthy that altered circadian rhythms of cortisol have been also found in autism [70]. Furthermore, single nucleotide polymorphisms (SNPs) in core circadian clock genes have been associated with ASD, suggesting a genetic contribution to these altered circadian rhythms [71]. In addition, decreased diurnal melatonin levels have also been described [72,73]. In line with these melatonin abnormalities, sleep–wake rhythm disturbances are observed in 40 to 86% of children with autism [74]. More precisely, an increased sleep latency (i.e., time to fall asleep), a decrease in total sleep duration as well as nocturnal and early morning awakenings have been reported in this population [75,76,77].

Moreover, 6-sulphatoxymelatonin (6-SM) excretion was found to be negatively correlated with the severity of social communication impairments in individuals with autism [78,79,80,81,82]. Despite the major role of melatonin in neurodevelopment [83], the causal link between melatonin and ASD needs yet to be established. Studies have reported lower melatonin levels in parents of children with ASD [72]. A more recent study found significant lower 6-SM excretion rates in mothers of children with ASD compared to controls [84] and the authors suggest that lower melatonin levels during pregnancy might be one of the risk factors for ASD. In line with these authors, Tordjman et al. [85] trying to understand better how so many genetic disorders involving different chromosomes and genes can lead to a common phenotype of autism with similar cognitive-behavioral features, propose among several hypotheses that early melatonin abnormalities may be possible risk factors for developing autism. The serotonin-melatonin-oxidative stress-placental intersection might be an especially fruitful area of biological investigation [86]. Further studies are necessary to explore and test these hypotheses.

It is noteworthy that lower nocturnal melatonin levels have also been reported as the most common melatonin abnormalities found in patients with schizophrenia compared to healthy controls [80,81,82]. In addition, lower early morning (7:00–8:00 am) melatonin levels have been observed in schizophrenia [87,88,89,90]. Close relationships have been described by several authors [91] between ASD and early-onset schizophrenia (EOS defined by an onset before 18 years old). Given that relationships are reported between ASD and EOS with negative symptoms of schizophrenia (such as social withdrawal or catatonia), and between EOS and negative symptoms (including between childhood onset schizophrenia and catatonia [92]), it could be expected that lower melatonin levels would be particularly observed in EOS patients with negative symptoms as a biological dimension shared by schizophrenia and ASD. However, no correlations were found between melatonin levels and negative or positive symptoms of schizophrenia assessed using the SANS (Scale for the Assessment of Negative Symptoms), SAPS (Scale for the Assessment of Positive Symptoms), or PANSS (Positive and Negative Syndrome Scale) [93]. Further research is necessary to study more thoroughly melatonin levels in EOS with negative symptoms, especially with social withdrawal observed also in ASD.

### 3.2. Therapeutic Benefits of Melatonin Administration in ASD

Melatonin supplementation has been studied in ASD patients since 1993 [69] and is part of recent treatment consensus guidelines [94]. Several studies reported therapeutic benefits following nighttime administration of melatonin for decreasing sleep latency as well as improving sleep duration and night awakenings (Evidence level Ia) in individuals with autism despite the small number of subjects (for a review, see Tordjman et al. [69]). In addition, melatonin supplementation might have positive effects on autistic behavioral impairments as suggested by a meta-analysis [74] and some placebo-controlled studies (improvement of social withdrawal, rigidity, communication, stereotyped behaviors, or anxiety) [78,79,80,81,82]. However, these therapeutic benefits are not currently subject to guidelines, and it is difficult to test the specificity of the results regarding autism given the bias of intellectual disability.

### 3.3. Review of Clinical Studies of Melatonin Supplementation in ASD

#### 3.3.1. Therapeutic Benefits Based on Melatonin Dose and Formulation

Melatonin supplementation in children with ASD was studied in 26 clinical trials [95,96,97,98] from 1996 to 2017 (0.75 to 12 mg of oral melatonin), (see Tordjman et al. [69] for an extensive literature review). Only eight randomized placebo-controlled trials (which represents a total of 621 subjects) included at least 11 subjects [78,79,80,95,99,100,101,102]. The majority of trials used 3–12 mg IR melatonin [78,79,99,100,101]; two trials used 3–5 mg controlled-release formulations of melatonin (PR combined with IR) [80,100], and one trial used 2–10 mg pediatric PR melatonin minitablets (PedPRM) [95,102]. It is noteworthy that although some of these trials were escalation studies or titration studies, none of them was a dose-effect relationship study comparing separated groups of ASD individuals for each different dose of melatonin. Total Sleep Time (TST), Sleep Latency (SL), and number of Night Awakenings (NA) were assessed for all these trials. Except for Garstang et al. (*n* = 11) [78], melatonin effects were significantly different from placebo with a significant reduction of SL and increase of TST. Interestingly, both IR and PR formulations improved SL with clinically and statistically meaningful change. TST was improved significantly by PR melatonin, but to a lesser extent by IR melatonin (participants using IR formulation slept on average 22 min longer, but they woke up earlier, and the confidence interval excluded the 60-min value determined to be the minimum clinically relevant) [99]. The number of awakenings decreased significantly only when using PedPRM [102], and the longest sleep episode was also significantly increased when PedPRM administration was compared to placebo administration (participants using PedPRM formulation slept on average 57.5 minutes longer compared to 9.14 minutes with the placebo without earlier waking time) [95,102]. The use of PedPRM-specifically adapted to a better compliance in a pediatric ASD population due to its small size and odor- and taste-less formula— significantly improved TST (up to an average of 57 min, *p* = 0.03) and SL (average decrease of 40 min, *p* = 0.01) [78,99]. Moreover, the authors compared other sleep parameters with previous studies using the IR formulation [99]. They found that wake-up time was delayed by 5.4 min when using PedPRM compared to the placebo, whereas wake-up time was earlier by 16 min with the IR formulation (explained by phase advance of the circadian melatonin rhythm in response to IR formulation). The authors explained this difference by PR characteristics. Further clinical trials are needed to compare directly PR and IR formulations. Standard sleep parameters (TST, SL, and NA) as well as other less well assessed key parameters (such as wake-up time and longest sleep duration) should be assessed in the future to compare IR and PR formulations.

Only one of these studies reported significant improvement on the clinical global impression scale (CGI) following the administration of controlled-release melatonin (combining IR and PR formulations) [80]. In addition, besides improvement of the autistic behavioral impairments described previously in Section 3.2, improvement of child externalizing behaviors was observed after the administration of PedPRM. More precisely, an improvement of insomnia-related problems in children with ASD, particularly externalizing behaviors, such as hyperactivity or aggression, and subsequently quality of family life-including parental quality of life and sleep as well as parental satisfaction concerning the child’s sleep habits—have been reported in studies using PedPRM or IR formulation [99,102,103]. The results suggest that melatonin has therapeutic benefits on several behavioral variables.

However, some melatonin trials suggested a loss of response to treatment in individuals with high melatonin salivary concentrations [104]. The authors stated the hypothesis that slow melatonin metabolization related to a SNP variant of *CYP1A2* could lead to high melatonin levels with a loss of therapeutic benefits of melatonin supplementation. Future studies should address this question of potential efficacy loss in a subgroup of individuals with ASD.

#### 3.3.2. Current Knowledge on Melatonin Dose and Tolerability in Typical Development and ASD

##### Optimal Daily Dose of Melatonin in ASD

There is no real consensus on the optimal daily dose of melatonin for sleep disturbances in children with ASD. French TRU recommends titration until a 6 mg daily dose [105] of melatonin and, although unlicensed in the UK for use in children, the British National Formulary for Children recommends a 10 mg maximum daily dose in children “aged 1 month to 17 years” [106]. This lack of official recommendation highlights the potential clinical relevance of summarizing safety and dosing data from clinical trials and post-marketing authorization reports.

Maximal dose of melatonin administered in healthy adult volunteer trials was 100 mg IV single dose [41] and 240 mg oral IR daily dose [51]. No adverse effects, including no sedation, were described [41]. Concerning short-term tolerability, the Rossignol et al. meta-analysis of melatonin treatment in individuals with ASD [74] reported good tolerability (0.5 to 15 mg) with no serious adverse event (drowsiness, awakening, and excitement were mainly observed). This meta-analysis did not report increases in epileptic seizures. Even in a later trial using 12 mg IR melatonin in 19 children during 8 weeks [99], only one adverse effect (not specified by the authors) in the melatonin treatment group was attributed to the experimental procedure.

For long-term use, Gringras et al. [95] reported more somnolence in the melatonin treatment group (expected adverse event) and headache compared to the placebo treatment group after 13 weeks of treatment in ASD children 2 years of age and over. In the one-year follow-up of the same group of children, Maras et al. [102] reported adverse events following melatonin supplementation in 95 children with ASD, 72 of them completed all efficacy assessments after one year of treatment. Among them, 29% of the children received per day 2 mg PedPRM, 47% received 5 mg/day PedPRM, and 24% received 10 mg/day PedPRM. The study continued to follow these patients up to 2 years, and 74 patients completed 104 weeks of treatment [24]. To our knowledge, this study is the one in which melatonin was administered for the longest duration and at the highest dose in ASD children from 2 years of age. This trial did not encounter serious treatment-related adverse events and only described adverse melatonin-related effects for 18% of patients (mainly fatigue, mood variations, irritability, aggression, hangover, and somnolence). However, adverse effects were not categorized by dosing group. Regarding the question of age, melatonin was administrated to 100 children from 3 months to 21 years old without encountered side effects [107] and was used in several clinical trials from 2 years of age [80,95,101], including in official reports [95]. French authorities allow melatonin use from 6 years old (this age cut-off is mainly due to the risk of choking under 6 years old with the previous PR melatonin formula), but Australian authorities do not recommend long-term administration for children [108] and the US National Institute of Health (NIH) specifies that melatonin “appears safe for short-term use but we don’t know about its long-term effects” [109]. Although the absence of scientific data justifies the French age cut-off, a recent study [110] underlines that melatonin supplementation exposes children to higher melatonin levels than adults. In fact, CYP1A2 activities are much lower in children than in adults (75% less CYP1A2 in 3 to 12 months age and 55% lower from 1 to 9 years old). In addition, Kennaway et al. [110] claim that, based on animal data, this supplementation could have effects on other hormones requiring long-term endocrine data in children and adolescent population. Further research is required to study the potential effects of melatonin supplementation on hormonal secretion, puberty, and reproduction.

##### Status of the Melatonin Supplementation: Medication or Nutritional Supplement?

Melatonin is a natural compound present in animals and at low (typically ng/g-µg/g) levels in a range of vegetables. This latter characteristic reinforces its status as a nutritional supplement. In addition, because melatonin is found naturally, it cannot be patented by pharmaceutical companies. However, specific pharmaceutical (galenic) formulations and/or therapeutic indications can be patented. In the United States, the FDA has classified melatonin only as a nutritional supplement. In Europe, melatonin has usually both the status of a nutritional supplement and a drug, depending on the dose. However, in some European countries, melatonin remains strictly available by medical prescription, for example in Switzerland and in the United Kingdom. It is noteworthy that nutritional supplements do not meet the same quality standards as pharmaceutical preparations. A recent survey in the United States showed that the nutritional supplements containing melatonin displayed an actual amount of 17% to 478% of the labeled content and that 26% also contained additional serotonin (1 to 75 µg) [111]. Similar results examining actual levels of melatonin in nutritional supplement preparations have been obtained in a British study [55]. The results indicate that oversight of melatonin supplement manufacture is warranted, and strongly suggest avoiding the consumption of products purchased on Internet. A recent recommendation related to safety data was reported by the French National Health Food and Environmental Safety Agency (ANSES) [112] for melatonin as a dietary supplement without medical prescription. ANSES highlights expected adverse effects (general symptoms, such as headaches or cardiovascular problems such as tachycardia) and gastroenterological symptoms. Among unexpected adverse effects reported by ANSES and the literature, hepatic cytolysis induced by melatonin is questioned in two reports [113], and three other adverse effects are controversial: role in the onset of epileptic seizure, triggering asthma crisis, and endocrine effects. However, ANSES confirms that it is difficult to examine and assess the specific role of melatonin given that other ingredients and impurities are present in dietary supplements. Although these side effects should be known and considered as serious concerns by physicians, as far as we know, no major side effect has been described until now. However, they do suggest that melatonin administration should be controlled by a medical prescription rather than over-the-counter consumption, as recommended by ANSES [112].

### 3.4. Relationships between Pharmacokinetics and Clinical Effects

These previous studies, despite their methodological rigor, do not investigate endogenous melatonin variation within individuals with ASD and do not correlate clinical effects and plasma concentrations after supplementation [43].

Very few studies have investigated both endogenous and exogenous melatonin PK profiles in an ASD population. In an open-labeled placebo-controlled PK-treatment study, Goldman et al. [97] collected endogenous melatonin data during placebo treatment administration and two weeks after the administration of IR melatonin liquid formulation in nine ASD children. They observed no difference in endogenous levels of melatonin compared to healthy volunteers for most of the children, contrary to previous data [69,72]. One of the hypotheses advanced is the difference in the analytical method for assaying melatonin. As for healthy volunteers, they noted a large baseline interindividual variability in endogenous melatonin (peak conc. from 42 to 310 pg/mL). Despite high interindividual variability, melatonin supplementation resulted in T_max_ and T_1/2_ (44 and 78 minutes) values with low interindividual variation. A delayed value for T_max_ compared to the available healthy volunteer data was observed. Although the observed T_1/2_ values were greater than those seen in healthy volunteers [47], they were close to tablet parameters. AUC for the group using 1 mg of oral melatonin was 4513 ± 3119 pg·h/mL and C_max_ was 2.505 ± 2.362 pg/mL. These standard deviations are explained by the authors to be due to variable hepatic first-pass effects. All patients showed improved sleep latency measured by actigraphy (*p* = 0.01) and night awakenings assessed by parental reports following melatonin supplementation. However, no significant improvement was observed for sleep duration (short effect duration) and other sleep parameters measured by actigraphy and polysomnography. This lack of improvement in sleep duration might be due in this study [46] to the administration of an IR melatonin formulation triggering a subsequent phase advance of the sleep–wake rhythm. Interestingly, among the five children for whom some improvement in the number of awakenings was reported, AUC values varied almost by a factor of 8 (from 1.377 to 10.137 pg·h/mL). In fact, in this cohort, the improvement in sleep latency and night awakenings was not correlated with C_max_ and AUC values. To explain this absence of a relationship between melatonin level and response, the authors hypothesized that supplemental melatonin could act by other mechanisms than “simply replacing melatonin”.

A second trial assessed PR melatonin PK profiles and tolerability as well as melatonin levels in 16 children with ASD (12 males and 4 females, 2–18 years of age). All of them completed the study. This was an open-label, single ascending dose study of 2 and 10 mg PR melatonin tablets [65,98]. It is noteworthy that melatonin was administered in the morning. Melatonin supplementation in children with ASD showed similar T_max_ values compared to healthy volunteers in PR formulations studies (1.5h) with an apparent half-life elimination of 5 hours and global linear PK despite wide interindividual variability. Based on the alertness/sedation scale, sedation (“drowsy/normal speech” level) was observed around T_max_ time for all patients, regardless of whether the dose was 2 or 10 mg. However, baseline melatonin profiles were difficult to interpret due to missing sampling points.

As previously indicated, very few clinical trials using melatonin have examined both PK and treatment effects Therefore, the concentration–effect relationship evidence needs to be investigated to understand the role of PK variability in clinical responses and explore if some PK parameters are correlated with improved clinical variables for sleep disorders as well as for autistic behavioral impairments. The clinical impact of both inter- and intraindividual variability must be investigated to understand if these differences are clinically relevant [43]. It might also be fruitful to look for formulations that avoid or minimize most of the hepatic first-pass effect. For example, the intranasal route has shown promising bioavailability results [114].

## 4. Measure of Melatonin Concentrations Depending on the Type of Sample and Analytical Methods in Healthy and ASD Individuals

### 4.1. Sample Type

Melatonin concentration is assessed to characterize endogenous circadian rhythms, variations of level with supplementation, and PK parameters. The type of sample must be taken into consideration, because studies do not always use the same samples or methods, which may add to the variability of the data. Thus, the sample type, the time and duration of collection, and the analytical method must be considered. Three different matrices are commonly used to estimate melatonin levels: blood, urine, and saliva (see Alves de Almeida et al. [115] for a detailed review). The main analytical methods used for melatonin level measurement in typically developing individuals are summarized in Table 2. The list of the studies presented in this table is not exhaustive.

#### 4.1.1. Blood Sampling

Blood sampling is generally the more informative and sensitive method. Guidelines [116] recommend a 20 to 30-minute sampling interval to evaluate the circadian phase of melatonin secretion under dim light conditions (Dim Light Melatonin Onset or DLMO), termination of melatonin synthesis (SYNOFF), peak plasma concentration, and other PK parameters, as well as total melatonin profile for 24 hours. Blood collection was used by Goldman et al. [97] and was well tolerated in nine drug-free prepubertal children with ASD (an intravenous catheter was placed early overnight, and all children were asleep during blood drawing). However, although blood (plasma) sampling is the preferred method particularly for individuals with low melatonin levels, this invasive method is not actually recommended for routine use [116]. Contrary to saliva and urine samples, it cannot be used at the patients’ home.

#### 4.1.2. Saliva Sampling

Saliva sampling is a usually reliable method requiring at least 0.4 mL saliva per tube taken every 30 to 60 minutes under dim light conditions. Saliva is particularly used for studying a melatonin 24 h profile or to determine DLMO. This method of sampling avoids the invasive procedure of blood sampling [80]. However, saliva melatonin levels are reported to be typically three times lower in saliva compared to blood. There is indeed a large body of evidence [53,116,117,118,119] showing a reliable and consistent 1:3 ratio between melatonin levels in saliva and plasma when sampled simultaneously, regardless of whether the source of melatonin was from endogenous production (low levels) or exogenous intake (high levels). This ratio was explained by Kennaway et al. [120], who established that melatonin saliva levels were highly correlated (*r* = 0.84) with free melatonin plasma levels (active fraction), but not with total melatonin plasma levels. It is noteworthy that melatonin saliva levels have been found in a more recent study [121] to be higher than plasma levels of free melatonin (on average 36% higher), which is possibly due to melatonin production in the salivary gland. In other words, comparing saliva sampling studies and blood (plasma) sampling studies can be complicated. Cavallo et al. [31] compared blood and saliva sampling in 33 volunteers with a range of ages and highlighted that the significance of protein binding is not clear for melatonin, but that binding protein variations could modify melatonin level measurement. However, Miles et al. [117,118] measured endogenous salivary and total plasma melatonin levels overnight at hourly intervals from 7 pm to 5 am in adult men and women and found that at each time point, the saliva to plasma melatonin ratio was 0.3 with a correlation coefficient close to 1.0 (*p* < 0.001 for all individuals); the 24-h profiles of plasma and salivary melatonin in healthy volunteers were very similar. Furthermore, Touitou et al. [119] reported a strong positive correlation (*r* = 0.97) between salivary melatonin levels and urinary excretion of 6-SM in children.

One practical limitation is that saliva sampling is not an easy procedure to perform in intellectually disabled children with ASD [70]. Awakening individuals with ASD for saliva collection can be difficult and burdensome for both parties [116]. In addition, saliva sampling has to be performed under controlled conditions, as brushing teeth that can provoke gum bleeding and food intake 20 min before sampling, as well as certain types of food (e.g., acidic food that increases salivary flow rate), may interfere with melatonin concentrations [70,122].

However, these studies taken together suggest that saliva sampling can be a useful method to implement with high-functioning individuals with ASD (children and adults). Salivary melatonin is a reliable marker of the melatonin circadian rhythm and has been widely used for the diagnosis and treatment of chronobiological disorders.

#### 4.1.3. Urine Sampling

As indicated previously, 6-sulphatoxymelatonin (6-SM) is the main metabolite excreted in the urine. Rates of 6-SM urinary excretion are well correlated with plasma melatonin levels (*r* > 0.7) and urine collection represents another non-invasive method [33,52,123]. Urinary 6-SM is used to estimate total melatonin production with a dim light sampling collection of all urine produced over periods of 2 to 12 hours, typically over a 24- hour period [116]. This method appears to be the most feasible one for melatonin level assessment in ASD but does require some degree of compliance. Urinary excretion can be expressed as amount per mg of creatinine or as amount excreted per unit of time. Whatever the type of collection, samples must be isolated from light to avoid melatonin degradation [115].

### 4.2. Sources of Analytical Variability

There are several analytical methods for the measurement of melatonin. In fact, although low levels of melatonin can be detected, there are only a few recent studies investigating analytical variability. Among the most widely used methods, immunological methods can present good sensitivity (limit of detection: 0.5 pg/mL), but a cross-reactivity risk exists [115]. Radioimmunological methods are available via different commercial kits with typical reported coefficients of variation (CVs) from 3.5 to 6.9% [124]. Radioimmunoassays (RIA) are validated for plasma, saliva, and urinary matrices measurement. For example, the RIA kit used by Andersen et al. [47] presented both inter- and intra-assay CV < 15% with a limit of detection of 2.3 pg/mL. There are also numerous enzyme-linked immunoassay (ELISA) commercial kits used for the analysis of melatonin in urine, blood, and saliva. A commercial ELISA kit was compared to the RIA method: a purification step (not specified by the manufacturer) was necessary to obtain good correlations with RIA for monitoring human blood melatonin [125]. In this study, the ELISA method presented a mean inter-assay CV of 15.4%, but it is more convenient than RIA (no radioactive wastes).

Promising results with liquid chromatography coupled with a tandem mass spectrometry (LC-MS/MS) method for plasma and saliva melatonin are now available (lower limit of detection under 1 pg/mL) with short analysis time and good inter- and intra-assay precision. The LC-MS/MS methods allow one to avoid the risk of cross-reactivity encountered in RIA or ELISA methods [116]. However, we are unaware of any direct comparisons between the LC-MS/MS and immunometric assays methods. Such studies would help in the interpretation of the variability seen in prior studies using the immunometric (RIA and ELISA) methods.

## 5. Conclusions and Perspectives

Melatonin represents a well-validated treatment for sleep disorders in children with ASD. However, there are to date insufficient data to determine the extent to which response variability has been dependent on interindividual exposure variability (PK and bioavailability effects). New therapeutic perspectives on melatonin are opening promising avenues for improving autistic behavioral impairments. Future clinical trials are necessary to examine therapeutic benefits of melatonin administration for autistic behavioral impairments based on dose-effect relationship studies. Finally, dose-effect relationship studies should take into consideration inter- and intra-individual variability factors of melatonin exposure to improve therapeutic effects of melatonin administration.

## Figures and Tables

**Table 1:** Main pharmacokinetic characteristics of melatonin administration in typically developing individuals based on the administration route and pharmaceutical form of melatonin.

Studies	Formulation Type	Pooled Number of Subjects	Daily Dose (mg)	Mean Bioavailability (Standard Deviation: SD)	Mean T_max_ in min (SD)	Mean T_1/2_ in min (SD)
Andersen et al., 2016 [41] DeMuro et al., 2000 [46] Fourtillan et al., 2000 [47] Andersen et al., 2016 [48] Mallo et al., 1990 [49] Le Bars et al., 1991 [50]	Intraveinous	60	0.005–100	-	Immediate (bolus)	42 (9)
Fourtillan et al., 2000 [47]	Oral suspension	12	0.25	12% (0.11)	23 (8)	40 (6)
De Muro et al., 2000 [46] Markantonis et al., 2008 [51]	Immediate-release tablet	22	2–6	15% (0.7)	49 (16)	56 (3)
Andersen et al., 2016 [48] Waldhauser et al., 1984 [52] Zhdanova et al., 1998 [53]	Immediate-release capsule	30	0.3–240	3% (Not available)	46 (3)	49 (4)
European Medicines Agency, 2007 [42]	Prolonged-release tablet	8	2	Not available	96 (48)	306 (120)

**Table 2:** Summary of the main analytical methods used for melatonin level measurement in typically developing individuals.

Studies Number of Individuals		Analytical Method	Sample	Measurement Duration (Hours)	Time of Administration/Collection
Waldhauser et al., 1984 [52] Mallo et al., 1990 [49] Le Bars et al., 1991 [50] Cavallo et al., 1996 [31] Zhdanova et al., 1998 [53] De Muro et al., 2000 [46] Härtter et al., 2000 [60] Ursing et al., 2005 [57] Tordjman et al., 2005 [69] EMA, 2007 [42] Tordjman et al., 2012 [74] Andersen et al., 2016 [41] Andersen et al., 2016 [48]	8 10 1 33 36 12 5 8 88 8 26 12 1	RIA	Blood/Urine Blood Blood Blood/Saliva/Urine Blood/Saliva Blood Blood Blood Urine Blood Urine Blood/Urine Blood	36 2 NA 6/6/12 24 8 28 6 12 24 24 7 7	11 am, 12 am, 1 pm 10 am NA 10 am 9 am 7–9 am 10 am From 8 am to 8 pm From 8 pm to 8 am 10 am 8 pm day1—8 pm day2 8 am 8 am
Fourtillan et al., 2000 [47]	12	GC-MS	Blood	13	10 am
Markantonis et al., 2008 [51]	18	HPLC-Fluorescence	Blood	5	8 am
Goldman et al., 2014 [97] (nanoflow LC–MS/MS for endogenous melatonin)	9	LC-MS	Blood	8	30 min before bedtime

Note: RIA: Radio Immunoassay; GC-MS: Gas Chromatography coupled with Mass Spectrometry; HPLC: High Performance Liquid Chromatography; LC-MS: Liquid chromatography coupled with Mass Spectrometry; LC-MS/MS: Liquid Chromatography coupled with Tandem Mass Spectrometry; EMA: European Medicines Agency; NA: Not Available.
